# Unlocking the full potential of rare disease drug development: exploring the not-for-profit sector’s contributions to drug development and access

**DOI:** 10.3389/fphar.2024.1441807

**Published:** 2024-08-12

**Authors:** Stefano Vavassori, Sean Russell, Celeste Scotti, Stefano Benvenuti

**Affiliations:** Fondazione Telethon ETS, Milan, Italy

**Keywords:** drug developement, NGO = nongovernmental organization, rare disease (RD), drug access, authorities, gene therapy (GT)

## Abstract

This commentary provides a comprehensive overview of the challenges and opportunities in the field of drug development for rare diseases and especially of gene therapy products for ultra-rare diseases. It discusses the limited market size, reimbursement and scientific complexities that deter pharmaceutical investment in this field. Highlighting the pivotal role of charitable organizations like Fondazione Telethon, it showcases their efforts in funding research and ensuring access to innovative therapies. This commentary also addresses the challenges in therapy distribution, particularly regarding sustainability and global access. It outlines Fondazione Telethon’s operational model to try to address these challenges. Finally, it appeals to governments and regulatory bodies to implement policies and incentives aimed at further fostering innovation and accessibility in rare disease drug development and access.

## Introduction

Rare Diseases (RDs) affect a small number of people compared to the general population. There is no single, widely accepted definition for RDs. For example, in the US, RDs are defined as those affecting fewer than 200.000 persons in the US (source: The Orphan Drug Act (ODA)), while in Europe, a disease is rare when it affects no more than one in 2000 people (Regulation, 1999). In other jurisdictions, for example, in Japan, low prevalence is not enough to secure the orphan drug status; the therapy should intend to treat a disease that affect less than 50.000 patients in Japan AND “for which there is an high medical need” (Source: Overview of Orphan Drug/Medical Device Designation System). It is estimated that 30 million people in the US (Andreu et al., 2023), 36 million people in EU, and around 400 million globally are affected by more than 7,000 RDs (80% of those are genetic) (source: Global Genes). Half of those affected by rare diseases are children (3 out of 10 children with a RD won’t live to see their fifth birthday (source: Global Genes) As many as 95% of RDs have no specific treatment or curative options (Pearson et al., 2022). In the US, a very recent report (Andreu et al., 2023) revealed that for a sample of 24 RDs the average overall cost to society is $266,000 PPPY (Per Patient Per Year) which is approximately 10x the cost associated with mass market diseases ($26,000 PPPY). RDs impose a substantial economic burden on society that is reduced by treatment availability (Andreu et al., 2023). In addition to having enormous economic and societal burdens, the drug development and distribution landscape for treatments of RDs is fraught with economic and regulatory complexities. In this context, charities, such as *Fondazione Telethon ETS* (http://www.telethon.it/en/) (FT), have been taking bold steps to offer innovative solutions (Monaco and Faccio, 2017). This commentary will explore how FT has played a crucial role in funding research and subsequently in securing access to a gene therapy for ADA-SCID patients, emphasizing the urgent need for the policy landscape to evolve to address the unique set of challenges in the RD’s field.

## A closer look at the lack of investment in rare disease therapies

**FIGURE 1:**
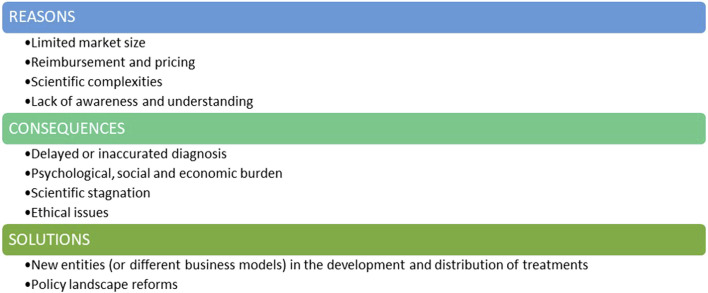
The lack of interest and investment in the genetic rare diseases field.

There appears to be a series of economic, scientific and ethical challenges currently influencing the investment landscape for rare diseases (Figure 1).

∙ *Limited marked size*: One of the main challenges when considering investments in therapies for rare genetic diseases is the limited patient population. Small market sizes may be less commercially viable due to the inability to deploy economies of scale. Most RDs fall into the category of ultra-rare and hyper-rare diseases, often affecting fewer than 100 people worldwide (Smith et al., 2022; Crooke, 2021). While conditions like cystic fibrosis or spinal muscular atrophy exemplify efforts to overcome market size constraints, the majority of RDs remain neglected due to their ultra-rare nature.
∙ *Reimbursement and pricing* can be a significant barrier. Even when a RD treatment reaches the market, challenges persist in terms of market access and pricing. Setting a fair price for RD’s treatment is an activity to be weighed carefully: on one hand, pharmaceutical companies need to cover development costs and generate revenue to payback investors and sustain further research, whilst on the other hand, high prices can raise ethical concerns and limit patient access, especially when healthcare systems face budget constraints. Uncertainty in the likely acceptable price-point and potential for return on investment can discourage initial investment and acceptance of the development risks.
∙ *Scientific complexity:* Many rare genetic diseases have poorly understood or complex pathophysiological mechanisms. The limited knowledge of disease progression and affected cellular pathways add significant complexity and creates an initial barrier to drug development. Genetic mutations and their diverse impacts lead to substantial variability in clinical presentations, even among patients with the same rare disease. Conducting initial clinical trials for these drugs is especially challenging due to the small pool of eligible patients, making recruitment time-consuming and costly. This financial burden can significantly deter investment in the rare disease field.
∙ *The lack of awareness and understanding* surrounding genetic RDs contributes to the limited attention they receive. Public awareness and advocacy are crucial in breaking this cycle of neglect. When communities are well-informed about RDs, they become powerful advocates for increased support and funding. This advocacy creates urgency among policymakers and funders to prioritize research and allocate resources to these often-overlooked conditions. Without widespread understanding of the impact of RDs, the challenges in addressing these diseases remain unmet.

The lack of interest and investment in this field carries profound consequences (Figure 1).

∙ One major consequence is *delayed or inaccurate diagnosis.* With limited research and awareness, healthcare professionals may struggle to recognize and understand these rare genetic conditions. This results in delayed or misdiagnoses, preventing timely intervention and appropriate management. Therefore, individuals with RDs may endure prolonged suffering, exacerbating the impact on their health (Faye et al., 2024).
∙ *Psychological, Social & Economic Burdens:* Individuals with RDs endure chronic pain, debilitating symptoms, and often a shortened lifespan. The lack of effective treatments exacerbates their suffering and diminishes their quality of life. This not only strains familial relationships but also places a heavy burden on healthcare systems and social support networks. Caregivers face significant costs, with many families seeing at least one parent forced to leave their job to care for a child with an RD.
∙ *Scientific Stagnation:* Neglecting RDs hampers scientific progress. Every RD is a unique genetic puzzle, and understanding these conditions could unveil insights into human biology and genetics, and physiology (Boycott et al., 2013; Lee et al., 2020). Discoveries made in the pursuit of treatments for these medical conditions have contributed, and can still contribute, to advancements in related fields, potentially unlocking innovative solutions for more common diseases.
∙ *Ethical Issues:* At the heart of ethical concerns is the principle of justice and equity, which asserts that every individual, irrespective of the rarity of their medical condition, should have access to the potential benefits of medical advancements. When certain conditions are neglected or overlooked due to a lack of interest or investment, it creates a disparity in healthcare accessibility. It is fundamentally imperative to ensure that the benefits of medical advancements are distributed equitably among all members of society, irrespective of the prevalence of their health conditions.

Solutions must be found and implemented: in addition to pharmaceutical companies, new entities may consider playing a pivotal role in the development and commercialization of RD treatments, and governments and regulatory agencies should implement reforms in their policies to unlock further investment in this critical area (Figure 1).

## Fondazione Telethon’s approach to this challenge

Established in 1990 in Italy, FT, a non-profit organization, emerged in the landscape of medical research with a mission to find a cure for each rare genetic disease and to make them all accessible to patients. Unlike pharmaceutical companies, FT’s is not profit-driven and its strategy is inspired by the needs of individuals affected by these often-neglected genetic conditions.

Since its inception, FT invested almost €700 Million in biomedical research in Italy in around 3,000 projects, and 637 genetic RDs studied through targeted grant programs, within two research institutes, *San Raffaele Telethon Institute for Gene Therapy* (SR-TIGET) in Milan, Italy, and *Telethon Institute of Genetics and Medicine* (TIGEM) in Naples, Italy. This, in turn, has enabled additional investments from pharmaceutical/biotech companies into these projects and institutes.

### Maintaining access to gene therapy for ADA-SCID: A critical decision for FT

ADA-SCID is a form of SCID (Severe Combined Immunodeficiency) caused by adenosine deaminase (ADA) deficiency (Flinn and Gennery, 2018). It is characterized by lymphopenia and extremely low immunoglobulin levels of all isotypes resulting in severe and recurrent opportunistic infections. ADA-SCID, often referred to as the “bubble boy” disease, is a rare genetic disease, and its annual incidence is estimated to be between one to five in 1,000,000 live births (source: Orphanet database).

In the 2000s, scientists of SR-TIGET developed an *ex-vivo* gene therapy that, with a single administration, corrects the genetic defects causing the disease (Migliavacca et al., 2024). In 2010 this program was out-licensed to GSK and finally, in 2016, EMA approved this therapy, called Strimvelis^®^. Soon after, GSK transferred the marketing authorization to Orchard Therapeutics that distributed the therapy until 2022, when it announced its intention to discontinue its investment in rare primary immune deficiencies (Press release Orchad Therapeutics, March 30th, 2022). The divestment impacted Strimvelis^®^ but also a developed gene therapy for the treatment of Wiskott-Aldrich syndrome (WAS) then labeled OTL-103. This *ex-vivo* gene therapy for WAS has also been originally developed by SR-TIGET. Since this divestment decision, FT has been playing a crucial role in committing to maintain Strimvelis^®^ accessible to patient and to move further in the development of the promising gene therapy for WAS now relabeled TLT003. This resulted, following a positive opinion from EMA in 2023 (source: EMA website, Strimvelis Procedural steps taken and scientific information after the authorisation), the marketing authorization for Strimvelis^®^ was fully transferred to FT granting therefore the access to this treatment for eligible ADA-SCID patients. The experience of Strimvelis^®^ represents an extreme example of the complexity of developing and distributing therapies for rare diseases as it sums the typical challenges of therapies for ultra-rare diseases with those of cell and gene therapies. However, as most of the RDs are genetic in origin and ultra-rare (Nguengang Wakap et al., 2020), Strimvelis^®^, being a gene therapy for an ultra-rare disease, exemplify a case that might become more and more common.

The challenges in distributing a treatment such as Strimvelis^®^ intended to treat patients with such a RD, are multifaceted.

∙ *Technology Limits:* The development of RD therapies takes many years and uses the most suitable available technology at the time of development. As an example, in this case the nature of the technology involved in manufacturing the therapy (*ex-vivo* gene therapy authorized only in a fresh formulation) limits the potential for cost reduction through economies of scale. Strimvelis^®^ has a limited shelf life necessitating its administration at qualified treatment center (i.e., San Raffaele Hospital in Milan, Italy), close to where it is manufactured. Developing a cryopreserved version of Strimvelis^®^ requires additional investments, which would be hard to recover, considering the rarity of ADA-SCID.
∙ *Financial Hurdles*: EMA does not offer automatic discounts on maintenance fees, neither for non-profit developers who may not fulfil the small or medium sized enterprise requirements nor for treatments targeting ultra-rare diseases beyond the currently available incentives for orphan medicinal products. For example, the current annual fee for maintaining an authorized product approved by the EMA is €128,100. This is an example of one such administrative cost that must be borne by the authorization holder and on top of other direct costs of getting the treatment to the patient.
∙ *Global limited access:* Strimvelis^®^ has received approval for utilization within the EU and UK. As a result, patients outside the EU may encounter obstacles in accessing it due to challenges in obtaining approval from local payers to fund a treatment not endorsed in their respective jurisdictions. Moreover, as currently Strimvelis^®^ is exclusively administered in Milan, it is priced only in Italy. This further limits the distribution to other EU countries’ healthcare systems as Strimvelis^®^, not having undergone the local price and reimbursement negotiation, is not included in the so-called “basket of care” of other EU countries. For this reason, despite the existing EU regulations on social security (Reg. 883/04 and 987/09) and the cross-border healthcare directive (Directive 2011/24/EU), access for non-Italian EU patients remains complex and the is not automatically granted by their healthcare systems. Access is subject to a prior authorization by local/national health authorities so that patients’ rights to seek treatment in another EU country where the therapy is available remain inadequately recognized.

These limitations to patients’ access could be systematically overcame only with i) the continued development of the product and introduction of new technological advances such as transitioning to a cryopreserved formulation, ii) the qualification of other centers around the world for the administration of Strimvelis^®^, iii) the registration in other jurisdictions, and iv) a distributed commercialization network that negotiate the price and reimbursement in any single jurisdiction. Unfortunately, this idealistic plan to further improve potential patient access is financially limited by the extreme rarity of the disease making it virtually impossible for FT to improve this situation as a result of the financial, technical and educational investments that would be needed. Even with an estimate of treating 10 patients per year globally, if Strimvelis^®^ were to be administered across all 27 EU countries, the US, and Canada, each treatment center would, on average, handle a single patient once every 3 years. This frequency falls significantly short of the ideal scenario for building the requisite expertise in the distribution and management of a complex treatment.

In conclusion, for ADA-SCID therapy, FT played two pivotal roles, firstly in funding the initial academic research to discover the therapy and secondarily to continue to make it available as much as possible despite the challenges.

### The challenges for Wiscott-Aldrich Syndrome’s therapy

Building on the model developed for ADA-SCID, FT has been continuing its commitment to addressing RDs, including also the *ex-vivo* lentiviral based gene therapy for Wiskott-Aldrich syndrome (WAS) called TLT003. WAS is a rare primary immunodeficiency that causes bleeding problems and eczema in addition to susceptibility to infections (Blundell et al., 2010). The incidence of WAS has been estimated at less than one in 100,000 live births (Orphanet Database, 2024). For TLT003 FT is intending to complete the development and to apply for marketing authorization to bring to patients a promising gene therapy product currently not being pursued by pharmaceutical industry. Like for ADA-SCID, the private sector is showing little interest in developing treatments for this medical condition. In 2023, upon request by San Raffaele Hospital in Milan (Italy) where TLT003 is administered, this therapy was included by AIFA (the Italian Medicines Agency, the public institution responsible for the regulatory activity of pharmaceuticals in Italy) in the national Early Access Scheme under the Italian law 648/1996, for which eligible patients may continue to have access to therapy, in an attempt to bridge the timeframe between the development completion and a marketing authorization being granted.

### The Fondazione Telethon’s model: key enablers

**FIGURE 2:**
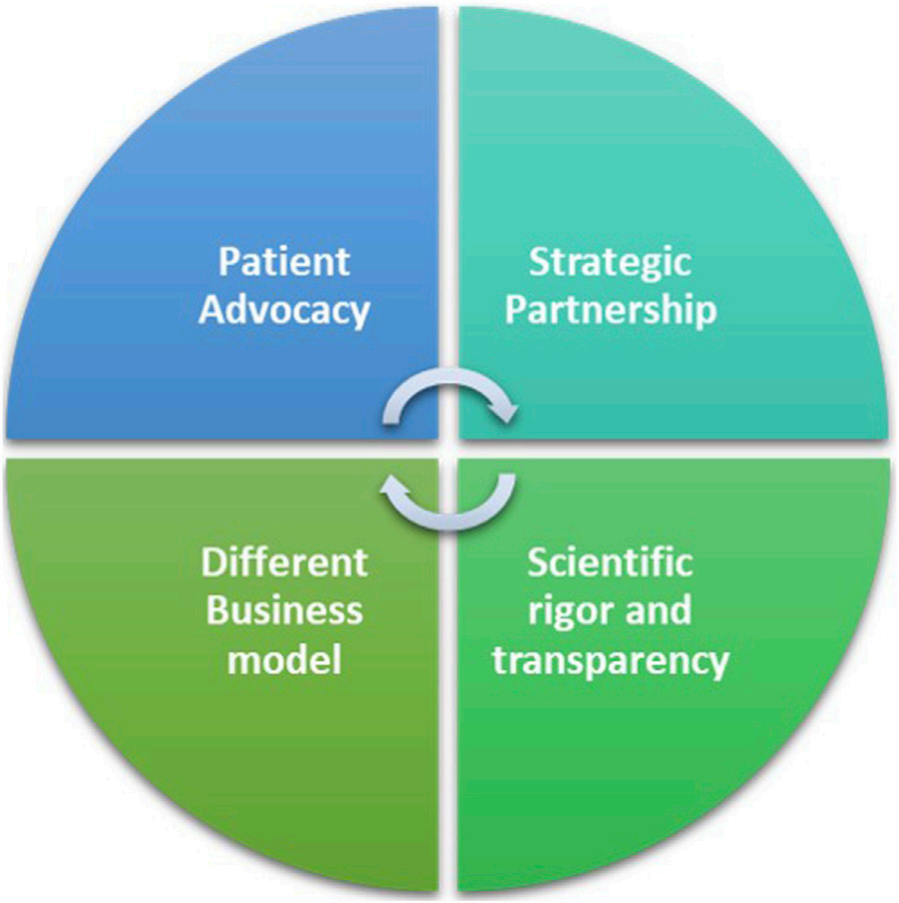
The Fondazione Telethon’s key factors for a workable alternative to the profit-driven model.

The FT model for ADA-SCID and outlook for WAS, can be attributed to a multifaceted approach that integrates rigorous yet flexible funding mechanisms, patient advocacy, strategic partnerships, and a distinct business model (Figure 2).

∙ *Scientific rigor and transparency in its funding scheme:* The FT peer review process is modelled on the system used by the National Institutes of Health, the US federal agency for biomedical research and it is Quality ISO9001 certified. The meticulous selection process underscores FT’s unwavering commitment to scientific excellence and innovation to identify research projects with the greatest potential to advance the understanding and treatment of rare genetic diseases (Jurkat-Rott and Lehmann-Horn, 2004; Pammolli et al., 2009).
∙ *Different business model:* While pharmaceutical companies have to consider market forces and shareholder returns alongside patients’ needs, FT’s can focus primarily on the latter. Its not-for-profit nature allows FT to prioritize impact over profit margins, ensuring that the needs of RD patients are at the forefront of decision-making.
   ⁃ FT’s reliance on donations and grants ensures a diverse funding base, reducing dependency on product profitability. In addition to philanthropic contributions, FT, as any other marketing authorization holder, obtains reimbursements by the healthcare system for Italian and European Union patients treated with the therapy it distributes (currently only Strimvelis). This financial structure helps maintaining affordability whilst ensuring FT sustainability and ultimately enabling the distribution of products in the market.
   ⁃ As a non-profit organization, FT has the ability to pursue projects that may be considered economically unviable by pharmaceutical companies. Traditional profit-driven models often shy away from endeavors with low profitability. However, FT’s flexible funding mechanisms allow it to invest in innovative projects and experimental therapies that have the potential to be transformative for patients no matter how profitable they will be.
   ⁃ FT do not need to generate a financial return on the original scientific investments as donors who fund the research activities of the organization do not ask for a financial return on their “investment”; what donors look for is the “societal benefit” produced by their money in terms of new treatments for rare diseases.
∙ *Patient Advocacy*: FT’s active involvement in patient communities stands as a cornerstone of its success. Through awareness campaigns, educational programs, and events, FT not only raises funds but also fosters a sense of solidarity and understanding among the public. In the realm of RDs, patients often find themselves marginalized, with limited visibility and advocacy. By involving patients in its initiatives, FT not only empowers the affected communities but also fosters a sense of urgency and commitment among stakeholders. This approach helps bridging the gap between the scientific community and those directly impacted by RDs, creating a shared mission that propels research forward.
∙   *Strategic Partnerships*: Collaboration lies at the core of FT’s strategy. Recognizing the complexity of RDs and the limitations of traditional pharmaceutical approaches, FT actively seeks partnerships with research institutions, biotech companies, service providers and academia. This collaborative approach allows FT to tap into diverse expertise and resources.

FT aspires to be a catalyst for change in the healthcare landscape, advocating for increased awareness, accessibility, and inclusivity in RD treatment. FT has not only pioneered groundbreaking treatments for overlooked conditions like ADA-SCID and WAS but has also presented an alternative model that operates independently of purely financial incentives. This model offers a viable non-competitive approach, alongside the profit-driven models crucial for the pharmaceutical industry. It's important to acknowledge the essential role of pharmaceutical companies in driving therapeutic innovation: their expertise, resources, and infrastructure are pivotal in bringing innovative treatments to market and ensuring global patient access. FT’s approach is not intended to replace the private model but rather to complement it, especially where profit-oriented frameworks may not be sustainable.

## Unlocking Investment in the rare diseases field: an appeal to governments and regulatory agencies

**FIGURE 3:**
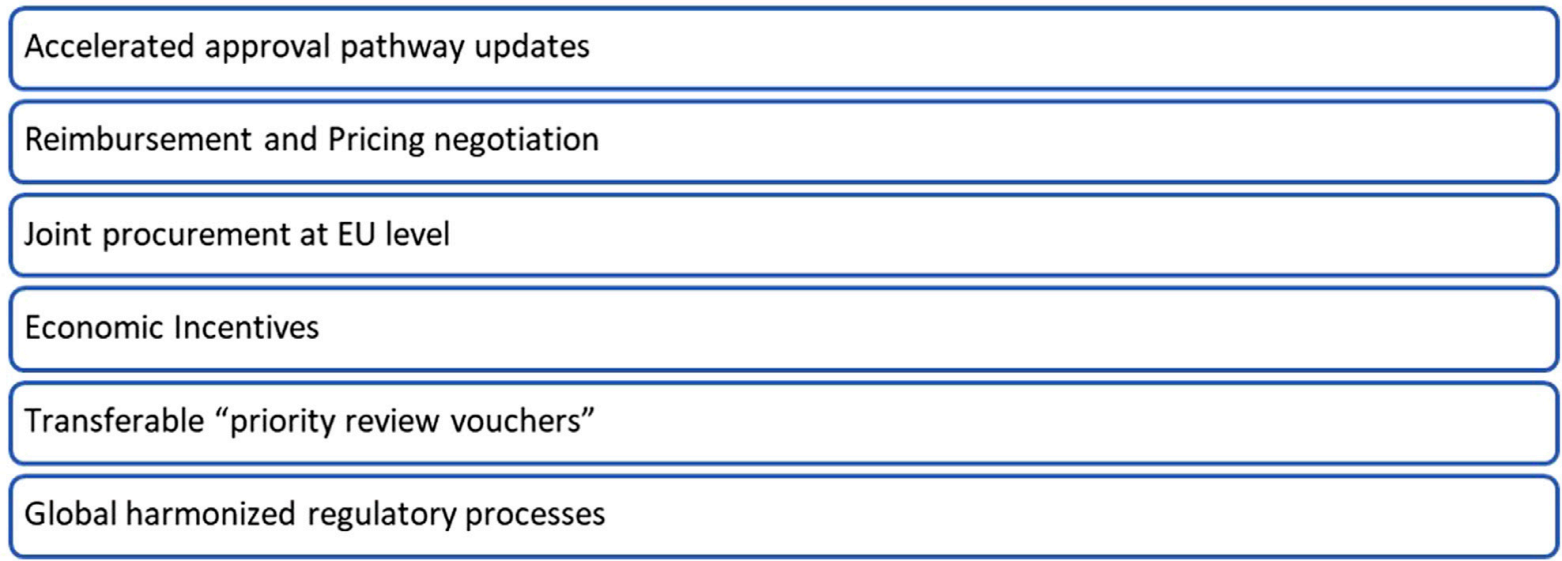
An appeal to governments and regulatory agencies.

Governments and regulatory bodies have made significant steps in incentivizing the development of orphan drugs, such as providing benefits of extended market exclusivity and accelerated approval processes. However, these incentives have limited utility for ultra- or hyper-rare diseases where the market competition is likely to limited and hence extended market exclusivity or earlier approval have limited true value. Pharmaceutical companies and charities still face the challenge of justifying investments in ultra-rare diseases. This highlights the need for strategies that enable drug developers to create, register, and distribute these drugs sustainably or to obtain or sustain financially viable rewards for the drug development investments made. Given the pivotal role of policy in shaping rare disease drug development, a strategic shift is essential. This commentary is not intended to be a compendium on economics, regulatory and policy reforms, it only provides the FT’s standpoint on them. Below are a few potential solutions to ensure both innovation and affordability (Figure 3).

∙ *Accelerated approval pathway updates*: Regulatory agencies should consider streamlining the drug approval process for RDs, with continued and further enhanced flexibility in clinical trial design and approval criteria. Adaptive licensing and conditional approvals allow for earlier market access while requiring post-approval data collection, thus furthering these approaches would recognize the potential for significant patient benefit earlier in the development pathway and hence enabling financial sustainability much earlier.
∙ *Reimbursement and pricing negotiation*: Adopting flexible pricing strategies, such as value-based pricing or instalment payment models, can address economic challenges and the unavoidable uncertainty in the evidence on long term efficacy available at the time of marketing authorization. This requires collaboration between drug developers and payers to develop sustainable pricing structures that ensure affordability without compromising innovation.
∙ *Joint procurement at the EU level* and EU-wide early access and cross-border healthcare programs to reduce the time to patients for centrally approved products (limited to ultra-rare diseases). The current market fragmentation necessitates individual negotiations for pricing and reimbursement across member states, resulting in several adverse outcomes: a) delay in access for patients; b) increase in the costs for managing the products; c) prolonged market entry timelines that mean a lower return on investment which indirectly makes the business case for these products further unfavorable. Recognizing the potential variance in prices among countries, influenced by differing willingness to pay and purchasing power, we advocate for a centralized pricing procedure that incorporates those factors. This approach would establish a 'purchasing power parity price,’ ensuring equitable access to all EU patients. Moreover, EU authorities might help by facilitating cross-border mobility of patients with ultra-RDs to access treatments in EU countries different from their country of residence.
∙ *Economic Incentives:* Governments should continue to offer incentives such as those associated with orphan drug designations, which grant exclusivity and financial benefits to organizations developing treatments for RDs. Governments can also provide financial incentives such as tax breaks, grants, or subsidies, to offset some of the development costs associated with RD drugs.
∙ *Transferable “priority review vouchers”* or analogous incentives, similar to the FDA Priority Review Voucher schemes, should be implemented within the EU framework for medicines addressing high unmet medical needs. This approach based on a “tradable” reward system for developers who manage to complete the registration of a product addressing high unmet medical needs could allow leveraging private “for-profit” funding to enhance the return on investment on “less profitable” products. In addition to revenue derived from product sales, such ventures could benefit from an additional one-off bonus income through the sale of these vouchers.
∙ *Global harmonized process*: Harmonization of regulatory requirements across regions is vital. Streamlining processes and aligning regulatory expectations can reduce costs and duplication of efforts and accelerate approvals. As an example, advanced therapy medicinal products are currently outside of the scope of EU/US mutual recognition agreements for inspections. For products under evaluation by both the FDA and the EMA, joint or mutual recognition of inspections could alleviate the burden and costs on the developers for these therapies.

These initiatives aim to encourage drug developers to invest in research for medical conditions that might not be financially attractive under conventional models.

## Empowering a global therapeutic ecosystem for rare diseases

**FIGURE 4:**
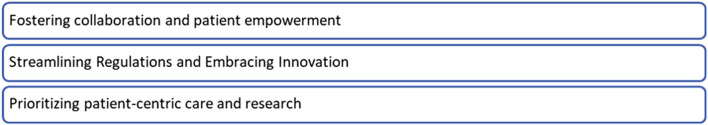
The key priorities to enhance patient outcomes and support.

Rare diseases pose significant challenges due to limited market incentives, complex regulations, and specialized expertise needs. While Europe (with EMA initiatives) and the US (with FDA programs like the Orphan Drug Act and Priority Review Vouchers) have made strides, the path to effective treatments remains arduous. To support the rare disease therapeutic ecosystem and improve patient outcomes, we prioritize three key areas (Figure 4).

∙ Fostering collaboration and patient empowerment

Strengthening international partnerships between research institutions, pharmaceutical companies, and patient advocacy groups is crucial. This includes fostering public-private partnerships and leveraging philanthropic contributions to accelerate breakthroughs.

• Global Partnerships: Initiatives like the TREAT-NMD Alliance and the Innovative Medicines Initiative (IMI) exemplify successful collaborations. The TREAT-NMD Alliance, a global network of patient advocacy groups for neuromuscular disorders, collaborates with researchers and pharmaceutical companies to accelerate clinical trials for new treatments (source: TREAT-NMD Alliance). They partnered with PTC Therapeutics to develop Translarna™, a therapy for Duchenne muscular dystrophy. IMI, a public-private partnership between the European Union and the European Federation of Pharmaceutical Industries and Associations (EFPIA), supports collaborative projects for rare diseases such as the *conect4children* (c4c) project which promotes innovation in the design of paediatric clinical trials to foster the development of new medicines in rare paediatric diseases (source: conect4children).
• Patient voices at the table ***:*** Patient advocacy groups play a vital role in raising awareness about the challenges faced by rare disease patients. Empowering patient advocacy groups like NORD (National Organization for Rare Disorders) in US and EURORDIS (Rare Diseases Europe) in Europe is crucial for raising awareness and influencing policies. Their efforts have led to significant advancements, such as the US Orphan Drug Act and the creation of European Reference Networks (source: rarediseases). Importantly, the International Rare Diseases Research Consortium (IRDiRC) serves as a global platform facilitating collaboration between research funding organizations, pharmaceutical companies, and patient advocacy groups. This multi-stakeholder approach is essential for accelerating research and development for rare diseases (source: IRDiRC - International Rare Diseases Research Consortium).

∙ Streamlining regulations and embracing innovation

• Harmonized regulations**:** As discussed in the previous chapter, advocating for streamlined and unified regulatory frameworks across different regions for orphan drugs would expedite patient access to life-saving treatments. This would also reduce costs for companies developing these specialized therapies.
• Leveraging technologies**:** Initiatives like PaVe-GT, led by the NIH, aim to streamline gene therapy development by standardizing platforms and protocols. By utilizing a common adeno-associated virus (AAV) vector for multiple rare diseases, PaVe-GT aims to reduce development timelines and costs significantly (source: NIH, NCATS). This approach, coupled with the rise of groundbreaking technologies like CRISPR-Cas9 gene editing, holds great promise for rare disease treatment. The FDA has approved CRISPR’s gene-editing therapy Casgevy^®^ to treat Sickle cell disease. Casgevy^®^ is the first FDA-approved therapy utilizing CRISPR/Cas9 (source: FDA website).

∙ Prioritizing patient-centric care and research

• Holistic approach to patient care: This encompasses ensuring accurate and timely diagnosis, facilitating access to new treatments, and providing ongoing support services for patients and families. For example, the NIH in the US established the Rare Diseases Clinical Research Network (RDCRN) to improve clinical research for rare diseases (source: Rare Diseases Clinical Research Network). The RDCRN connects patients with clinical trials and provides resources to help them participate in research.
• Investing in natural history studies: Creating comprehensive natural history studies and patient registries is crucial for understanding the natural course of rare diseases. Natural history studies track the course of a disease in untreated patients, providing invaluable insights into disease progression, symptom variation, identify potential treatment options and potential complications. This knowledge is the solid foundation for crafting effective treatments, identifying predictive biomarkers, and optimizing clinical trials. For instance, the Huntington Study Group’s longitudinal research has been instrumental in defining disease stages and developing outcome measures for therapeutic interventions (source: The Huntington Study Group). Similarly, the Natural History Study of Fabry Disease, conducted by the Fabry Support and Awareness group, has provided critical insights into the disease’s progression and the impact of early intervention (source: FSIG website). Beyond disease trajectory, natural history studies contribute to understanding patient experiences, identifying unmet medical needs, and informing public health policies.

## Navigating the challenges and charting the future of the rare disease drug development

The lack of interest and investment in RDs and especially in ultra-rare diseases carries profound consequences, impacting not only the affected individuals and their families but also impeding scientific progress, ethical standards, and sustaining an economic burden for society. While progress has been made, some challenges remain in the RD drug development and commercialization lifecycle. Issues such as the suboptimal regulatory landscape and ensuring fair access to treatments are still on the agenda.

The FT model operates in harmony with traditional pharmaceutical business models, filling gaps that may not attract private sector investment, and offering hope and real solutions for patients who may otherwise be overlooked. This model serves as a blueprint for a complementary approach to RD drug development and distribution, potentially revolutionizing the field. To improve the rare disease therapeutic ecosystem and support patients’ lives, we must focus on fostering international collaboration, streamlining regulatory processes, prioritizing patient-centric care and research. By addressing these priorities, we can build a more responsive, efficient, and patient-centered ecosystem for rare disease therapeutics. Only through collective action can we unlock the full potential of therapeutic interventions for rare diseases and improve outcomes for those in need.
